# Oxford nanopore long-read sequencing enables the generation of complete bacterial and plasmid genomes without short-read sequencing

**DOI:** 10.3389/fmicb.2023.1179966

**Published:** 2023-05-15

**Authors:** Wenxuan Zhao, Wei Zeng, Bo Pang, Ming Luo, Yao Peng, Jialiang Xu, Biao Kan, Zhenpeng Li, Xin Lu

**Affiliations:** ^1^National Institute for Communicable Disease Control and Prevention, Chinese Center for Disease Control and Prevention, Beijing, China; ^2^Department of Health Statistics, School of Public Health, Shanxi Medical University, Jinzhong, Shanxi, China; ^3^School of Public Health, Shandong University, Jinan, China; ^4^Yulin Center for Disease Control and Prevention, Yulin, Shanxi, China; ^5^School of Food and Chemical Engineering, Beijing Technology and Business University, Beijing, China

**Keywords:** ONT sequencing, accuracy, complete genome, bacteria, plasmid

## Abstract

**Introduction:**

Genome-based analysis is crucial in monitoring antibiotic-resistant bacteria (ARB)and antibiotic-resistance genes (ARGs). Short-read sequencing is typically used to obtain incomplete draft genomes, while long-read sequencing can obtain genomes of multidrug resistance (MDR) plasmids and track the transmission of plasmid-borne antimicrobial resistance genes in bacteria. However, long-read sequencing suffers from low-accuracy base calling, and short-read sequencing is often required to improve genome accuracy. This increases costs and turnaround time.

**Methods:**

In this study, a novel ONT sequencing method is described, which uses the latest ONT chemistry with improved accuracy to assemble genomes of MDR strains and plasmids from long-read sequencing data only. Three strains of Salmonella carrying MDR plasmids were sequenced using the ONT SQK-LSK114 kit with flow cell R10.4.1, and de novo genome assembly was performed with average read accuracy (Q > 10) of 98.9%.

**Results and Discussion:**

For a 5-Mb-long bacterial genome, finished genome sequences with accuracy of >99.99% could be obtained at 75× sequencing coverage depth using Flye and Medaka software. Thus, this new ONT method greatly improves base-calling accuracy, allowing for the de novo assembly of high-quality finished bacterial or plasmid genomes without the need for short-read sequencing. This saves both money and time and supports the application of ONT data in critical genome-based epidemiological analyses. The novel ONT approach described in this study can take the place of traditional combination genome assembly based on short- and long-read sequencing, enabling pangenomic analyses based on high-quality complete bacterial and plasmid genomes to monitor the spread of antibiotic-resistant bacteria and antibiotic resistance genes.

## 1. Introduction

Microbial resistance to drugs has become a global issue of widespread concern (Nathan, 2020). The widespread emergence of antibiotic resistance, especially multidrug resistance (MDR), among bacterial strains that cause infections presents difficulties in clinical treatment. Acquired resistance spreads very rapidly compared with chromosomal mutations (Paterson and van Duin, 2017; Bengtsson-Palme et al., 2018). Plasmids are the most common vectors for horizontal gene transfer (San Millan, 2018; Vit et al., 2020). In the concept of One Health, it is crucial to investigate MDR plasmids, because their domain organization is critical to the spread of antimicrobial resistance genes (ARGs) among bacteria (Aslam et al., 2021). Therefore, to track the transmission of ARGs, accurate information on MDR plasmid genomes is essential (Bennett, 2008; Malhotra-Kumar et al., 2016; Jordt et al., 2020).

MDR mostly originates from the accumulation of resistance genes on plasmids (Nikaido, 2009), though resistance genes can also be carried on the chromosome. Identifying these genes and their accurate genomic localization using short-read sequencing data can be difficult (Partridge et al., 2009). To associate independent data with ARGs transmission events, pangenome clustering based on complete plasmid genomes can be applied to surveillance (Li et al., 2022). Short-read sequencing [such as from Illumina and MGI next-generation sequencing (NGS) technologies] has high base-calling accuracy, but the nature of the short reads means that only fragmented draft genomes can be obtained from such data. Instead, scientists would prefer to receive correct, complete genomes as their study advances (Cohen et al., 2019; Kathirvel et al., 2021). Shortly after the introduction of NGS, third-generation sequencing technologies (TGS) emerged, presented by two platforms, Pacific Biosciences (PacBio) and Oxford Nanopore Technologies (ONT), giving long and ultra-long sequencing reads, respectively, and enabling coverage of highly repetitive regions and structural variants. PacBio developed the first established single-molecule real-time sequencing technology in 2011 (Athanasopoulou et al., 2021). In 2014, high-throughput, long-read sequencing was made possible by ONT on a portable device MinION (Lu et al., 2016). However, compared with NGS, low base-call accuracy has limited the reliability of ONT data for critical genomic epidemiology tasks (Petersen et al., 2019; Foster-Nyarko et al., 2023). The widely accepted remedy was to use short-read NGS data for error correction of long-read sequence data (Senol Cali et al., 2019; Smith et al., 2020).

In this study, our main objectives were to produce a high-quality finished genome only by *de novo* assembly based on long-read sequencing and to offer solid evidence for resistance gene analysis, supporting its use in the genome-based epidemiological analyses. This approach offered a more efficient and cost-effective replacement for traditional methods that required both long- and short-read sequencing. First, the reference genome of the strains was generated. Then, the sequencing data were obtained using the new ONT SQK-LSK114 kit with flow cell R10.4.1, and use of long-read sequencing data only for *de novo* genome assembly of these strains, combined with the error correction of the data itself. Finally, the accuracy of these genomes was verified against the reference data. To evaluate the accuracy of our method, we assessed the single nucleotide variations (SNVs), insertions (INSs), and deletions (DELs), which are common *de novo* assembly errors (Boostrom et al., 2022). To compare the new sequencing method with the earlier sequencing methods, all the samples were sequenced using the SQK-LSK110 kit and the R9.4 flow cell. In comparison with the previous version (Sereika et al., 2022), sequencing quality was substantially improved in the latest ONT chemistry and has potential implications for monitoring the spread of antibiotic-resistant bacteria and antibiotic-resistant genes.

## 2. Materials and methods

### 2.1. Samples

*Salmonella* strains were collected from the surveillance of healthy people, and we constructed a strain bank from these strains. Antimicrobial susceptibility testing was conducted and interpreted using the broth microdilution method recommended by the Clinical and Laboratory Standards Institute. Three MDR *Salmonella* strains were recovered from the strain bank and isolated with *Salmonella* agar (CHROMagar Company, Paris, France); strains were identified using the Vitek-II system (bioMérieux, Lyon, France). The genomic DNA from each strains was extracted by boiling and freeze-thawing processes, and the resulting supernatant was recovered for use as the PCR template (Doyle et al., 2012; Ding et al., 2020; Fan et al., 2022).

### 2.2. Sequencing

Genomic DNA was extracted using the TIANamp Bacteria DNA Kit (TIANGEN, China) and quantified using a Qubit V4 Fluorometer (Thermo Fisher, United States). Sequencing libraries were prepared using Ligation Sequencing Kit V14 (SQK-LSK114, ONT) and sequenced using R10.4.1 flow cells (FLO-MIN114, ONT) on a GridION device (ONT) with MinKNOW v22.08.9 and super-accuracy base-calling mode selected. Other parameters were kept at their defaults. To compare the new sequencing method used in our study with the earlier sequencing methods, we sequenced all the samples using the SQK-LSK110 kit and the R9.4 flow cell.

NGS libraries were constructed using the MGIEasy FS DNA Library Prep Set (MGI, China) and sequenced on the MGISEQ-200RS sequencing platform (MGI).

### 2.3. Data analysis

Guppy v6.2.11 (Wick et al., 2019) was used to extract the bases from the downloaded fast5 data and turn them into standard fastq files. NanoPlot v1.20.0 was used to assess the level of sequencing quality (De Coster et al., 2018). NanoFilt v2.8 (De Coster et al., 2018) was used to remove sequences that were <1,000 bp long with quality value < 10. In addition, 50 bp were removed from the front and back ends of each clean data record.

It has been widely used in earlier studies and proved to be the most accurate method to assemble reference genomes utilizing short- and long-read sequencing (Petersen et al., 2019; Boostrom et al., 2022; Sereika et al., 2022). In our study, we utilized this method to generate the reference genomes. Long-read data with 500 × depth and short read data with 500 × depth were used, and were assembled using the Unicycler hybrid assembler v0.4.8 (Wick et al., 2017). Pilon v1.24 (Walker et al., 2014) was used to polish these genomes. The obtained genomes were used as references for the following analyses.

Raw data packets were generated periodically during nanopore sequencing, typically at 6-min intervals (approximately 4,000 reads). The depth of coverage was based on packet size. After the clean data were generated, it was divided into depths: 1× (6 min), 5× (12 min), 10× (25 min), 20× (40 min), 30× (75 min), 50× (105 min), 75× (175 min), 100× (265 min), 150× (360 min), 200× (460 min), 250× (560 min), 300× (660 min), 350× (760 min), 400× (850 min), 450× (960 min), and 500× (1,050 min). The final depth of coverage was estimated based on the actual data size. The goal was to determine the saturation sequencing time point and depth.

**Figure 1:**
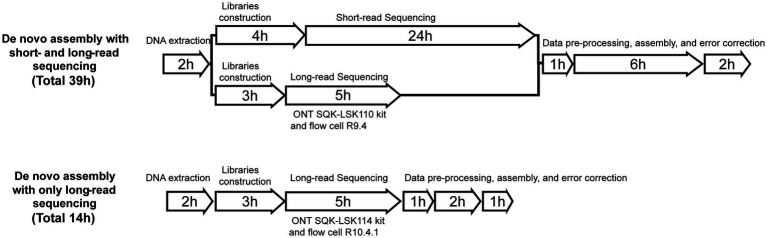
Time required to perform each method.

Flye has proven to be the most effective tool for *de novo* genetic assembly (Boostrom et al., 2022). Therefore, the default parameters of Flye v2.8.2 (Kolmogorov et al., 2019) were used for *de novo* assembly, and QUAST v5.2.0 (Gurevich et al., 2013) was used to evaluate the quality of genome assembly. Finally, errors were corrected by applying Medaka v1.2.2 three times.^1^ BLAST v2.11.0 was utilized to determine the identity of the fastq file in comparison to the reference genome. Snippy v4.4.5 was used to compare the assembled fasta file to the reference genome and obtain the number of SNPs, insertions, and deletions.^2^ ARG genes were aligned using the ResFinder database. FastANI v1.33 (Jain et al., 2018) was used to calculate genome-wide average nucleotide identity between genomes. R v4.1.0 and BRIG v0.95 (Alikhan et al., 2011) were used to visualize the outcomes (Figure 1). For detailed usage instructions, please refer to the Supplementary material.

The complete sequences of all three strains have been deposited in the GenBank database, Their BioProject ID is PRJNA937772.

## 3. Results

### 3.1. Characteristics of sequences

In total, we used three MDR *Salmonella* strains (SA15303, SA14318, and SA17155) that have been shown by PCR to carry the *mcr*, *ndm*, and *tet* genes, respectively. After hybrid genome assembly from long and short reads, we used short reads for multiple rounds of polishing. The aim was to obtain a high-quality reference genome for each strain. The complete genomes for strains SA15303, SA14318, and SA17155 were 5.1, 4.7, and 5.2 Mb long, respectively. The plasmids of these strains were 29, 199, and 287 kb in size, and contained 4, 9, and 12 ARGs, respectively. Thus, the plasmid size as well as the resistance genes varied widely between the strains. There were more homopolymer regions on the larger plasmids (Table 1).

**Table 1:** Details of the strains used in this study and their plasmids.

	SA15303	SA14318	SA17155
Total size	5,144,932 bp	4,742,137 bp	5,240,120 bp
Plasmid size	286,565 bp	29,276 bp	199,024 bp
Plasmid type	IncHI2A	IncX1	IncHI2
Number of resistance genes on the plasmid	9	4	12
Number of homopolymer regions on the plasmid	131	7	94
MDR profiles	Chloramphenicol-Gentamicin-Tetracycline-Trimethoprim-Sulfamethoxazole	Aztreonam-Cefazolin-Ceftazidime-Ceftriaxone-Chloramphenicol-Ciprofloxacin-rtapenem-Meropenem-Imipenem-Tetracycline-Sulfamethoxazole	Cefazolin-Ceftazidime-Ceftriaxone-Gentamicin Chloramphenicol-Ciprofloxacin-rtapenem-Meropenem-Imipenem-Tetracycline-Sulfamethoxazole

**Figure 2:**
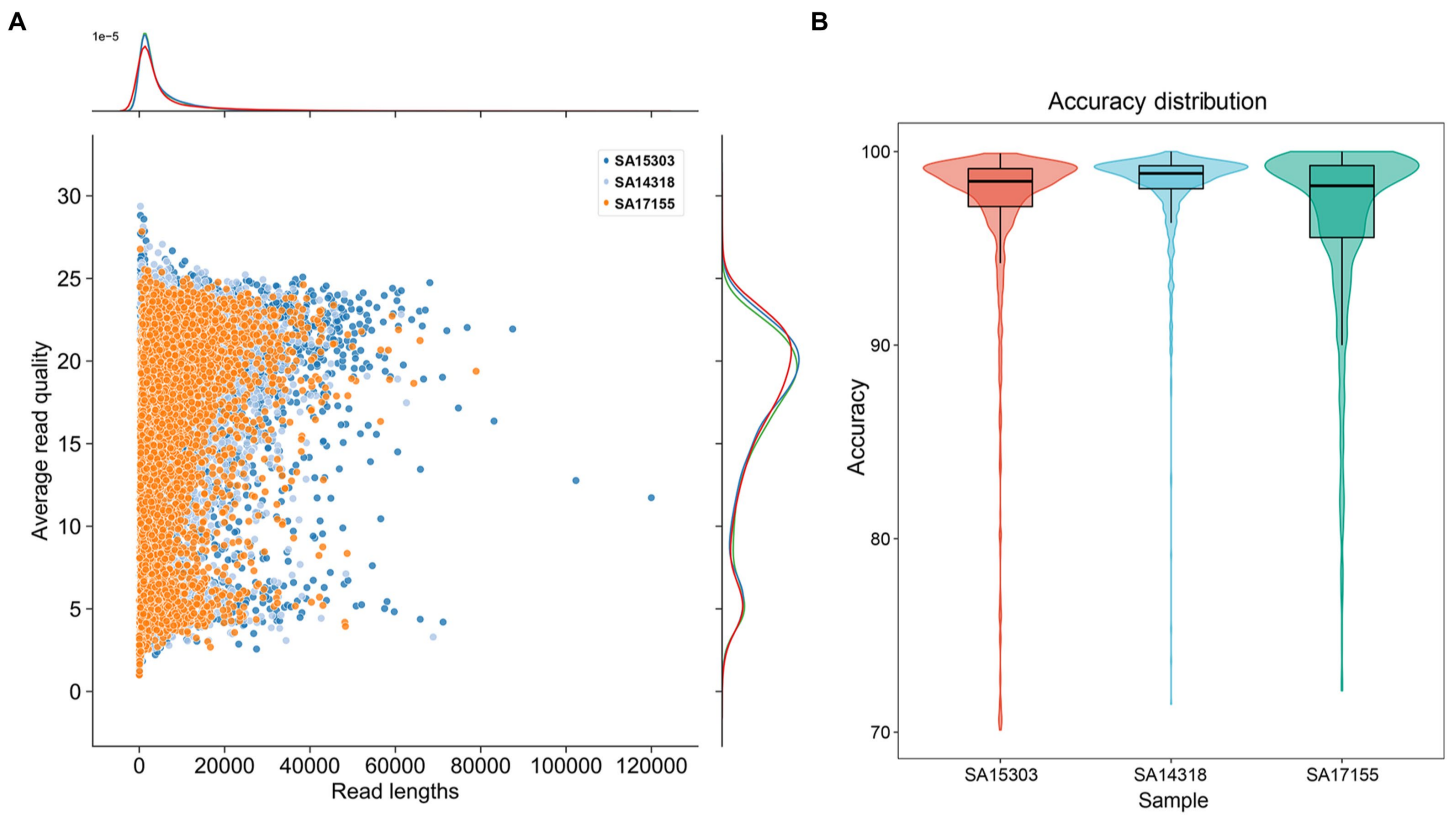
Summary of read quality. **(A)** The distribution of sequence length and sequence Q value of duplex data. **(B)** Box plot of read accuracy compared with reference data.

In experiments to test genome generation using only long-read sequencing data, we used a combination of the latest ONT kit v14 (SQK-LSK114) and flow cell R10.4.1 to obtain the raw data. After sequencing for 18 h, each flow cell generated about 500× data, with 39%–42% of the reads having lengths > 5 kb. Multiple reads of >60,000 bp were obtained (Figure 2A). Reads with quality value Q10 exceeded 85%, while those with Q20 exceeded 40% (Table 2).

**Table 2:** Summary of sequencing data.

		SA15303	SA14318	SA17155
Sample concentration (ng/μL)		97.2	95.2	96.2
SQK-LSK114 kit and flow cell R10.4.1	Total file size (gigabytes)	5.03	4.73	4.95
	Depth	508×	497×	519×
	Total reads	361,153	309,825	318,461
	Reads > 5 kbp (%)	42.06	39.34	46.15
	Average length of reads (kbp)	5.83	4.98	5.18
	Percentage of Q10(%)	88.27	85.58	87.2
	Percentage of Q20(%)	52.67	42.31	46.25
	Average accuracy of reads (%)	98.94	98.88	98.83
	Alignment error rate (%)	2.19	2.11	1.99
SQK-LSK110 kit and flow cell R9.4	Total file size (gigabytes)	5.05	4.87	4.89
	Depth	512×	512×	511×
	Total reads	656,556	457,263	504,270
	Reads > 5kbp (%)	28.53	33.15	37.22
	Average length of reads (kbp)	3.96	4.59	4.14
	Percentage of Q10(%)	89.74	85.26	82.52
	Percentage of Q20(%)	13.65	14.22	18.09
	Average accuracy of reads (%)	94.02	92.53	93.84
	Alignment error rate (%)	5.79	7.18	5.11

We used an identical procedure to sequence the genomes of the three strains. The read lengths and Q-score distribution of the three sets of sequence data were similar (Figure 2A). The average length was 5 kb, and the average Q value was 16.70. We considered a Q value < 10 to represent low-quality reads and removed these (approximately 14% of the whole sequencing data) before assembly. When compared with the reference genome sequences, the average read accuracy was 98.9% (Figure 2B), with the lower quartile > 95%.

To compare the new sequencing method with the earlier sequencing methods, all the samples were sequenced using the SQK-LSK110 kit and the R9.4 flow cell. It was important to note that the new sequencing technique shown considerable increases in both quality and accuracy. More specifically, accuracy increased from 92% to 98% and Q20 increased from 13% to 42% (Table 2).

### 3.2. Genome assembly and error correction

After removing the low-quality reads, *de novo* assembly was performed using Flye to obtain preliminary results. Medaka was then run three times to correct errors. About 10× coverage (100 Megabyte, 25 min) was able to obtain complete plasmid sequences for all three strains. However, to obtain the complete sequence of both chromosomes and plasmids, coverage needs to be increased to 30× (300 Megabyte, approximately 75 min; Table 3).

**Table 3:** The number of errors in chromosome and plasmid sequences.

		Running time (min)	25	75	175	1,050
SA15303	Chromosome	Coverage depth	9×	31×	75×	477×
		Whether cyclic	N	Y	Y	Y
		Size (bp)	1,199,135	4,848,505	4,848,501	4,848,495
		Errors	532	10	9	5
	Plasmid	Coverage depth	11×	41×	94×	594×
		Whether cyclic	Y	Y	Y	Y
		Size (bp)	286,566	286,565	286,567	286,564
		Errors	15	2	2	3
SA14318	Chromosome	Coverage depth	13×	39×	81×	450×
		Whether cyclic	N	Y	Y	Y
		Size (bp)	3,517,587	4,712,839	4,712,855	4,712,849
		Errors	123	11	8	9
	Plasmid	Coverage depth	6×	22×	55×	306×
		Whether cyclic	Y	Y	Y	Y
		Size (bp)	29,281	29,266	29,276	29,276
		Errors	19	0	0	0
SA17155	Chromosome	Coverage depth	11×	31×	71×	466×
		Whether cyclic	N	Y	Y	Y
		Size (bp)	3,256,928	5,031,756	5,030,613	5,030,601
		Errors	535	12	9	8
	Plasmid	Coverage depth	9×	23×	51×	358×
		Whether cyclic	Y	Y	Y	Y
		Size (bp)	199,056	199,024	199,022	199,023
		Errors	50	4	2	3

Compared with the reference genome sequences, the errors generated can be classified as DELs, INS, and SNVs. SNVs represented the largest number of errors (48%), followed by DELs (26%). When the depth of the sequencing data reached 75× (750 Megabyte, 175 min), the assembly error rate was stable, and we obtained satisfactory sequences (Table 3). After assembly, the error-correction capabilities of Medaka were clearly visible; with DEL errors being the majority. Among them, 83.33% of DEL errors were eliminated, and, subsequently, 68.42% of SNV errors were corrected (Table 4). Medaka tends to fix DEL errors, which were more likely to occur in *de novo* assembly. The initial error correction impact of Medaka was notable, whereas the second and third error correction effects had no appreciable improvement (Supplementary Table 1).

**Table 4:** Errors before and after correction.

	Type of variant	Before correction	After correction
SA15303	Variant-DEL	10	2
	Variant-INS	3	7
	Variant-SNP	3	2
SA14318	Variant-DEL	8	0
	Variant-INS	2	2
	Variant-SNP	7	5
SA17155	Variant-DEL	6	1
	Variant-INS	1	4
	Variant-SNP	9	6

**Figure 3:**
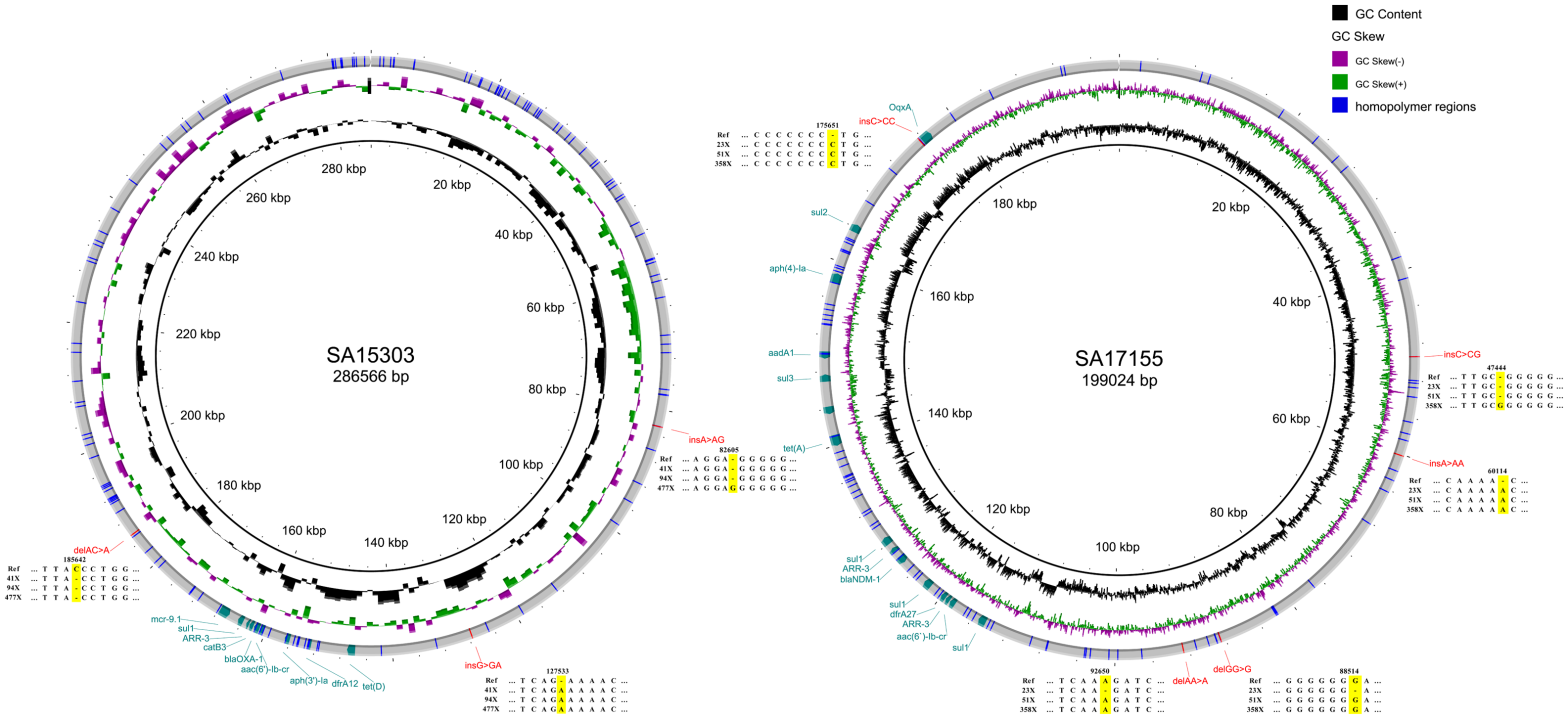
Plasmid structure diagrams for strains SA15303 and SA17155. Compared with the reference sequences, the errors at 30× coverage depth were showed. The plasmid of SA14318 has no errors and was not shown in this figure.

At a depth of sequencing data of 500×, the genomes of the three strains still had, respectively, 8, 9, and 11 errors that could not be corrected when compared with the reference genome sequences. Among SNV errors, G was frequently misidentified as T (8/9) and C was frequently misidentified as A (4/5). Duplication of the same base was the cause of insertion and deletion errors that could not be corrected. On chromosomes, 88.89% of the errors were found in homopolymer regions (Supplementary Table 2). Likewise, on plasmids, all INS and DEL errors were found in homopolymer regions (Figure 3). The reason for the above faults may be that Medaka is not adapted to the latest ONT model; thus, sequencing correction may improve with future software updates.

**Figure 4:**
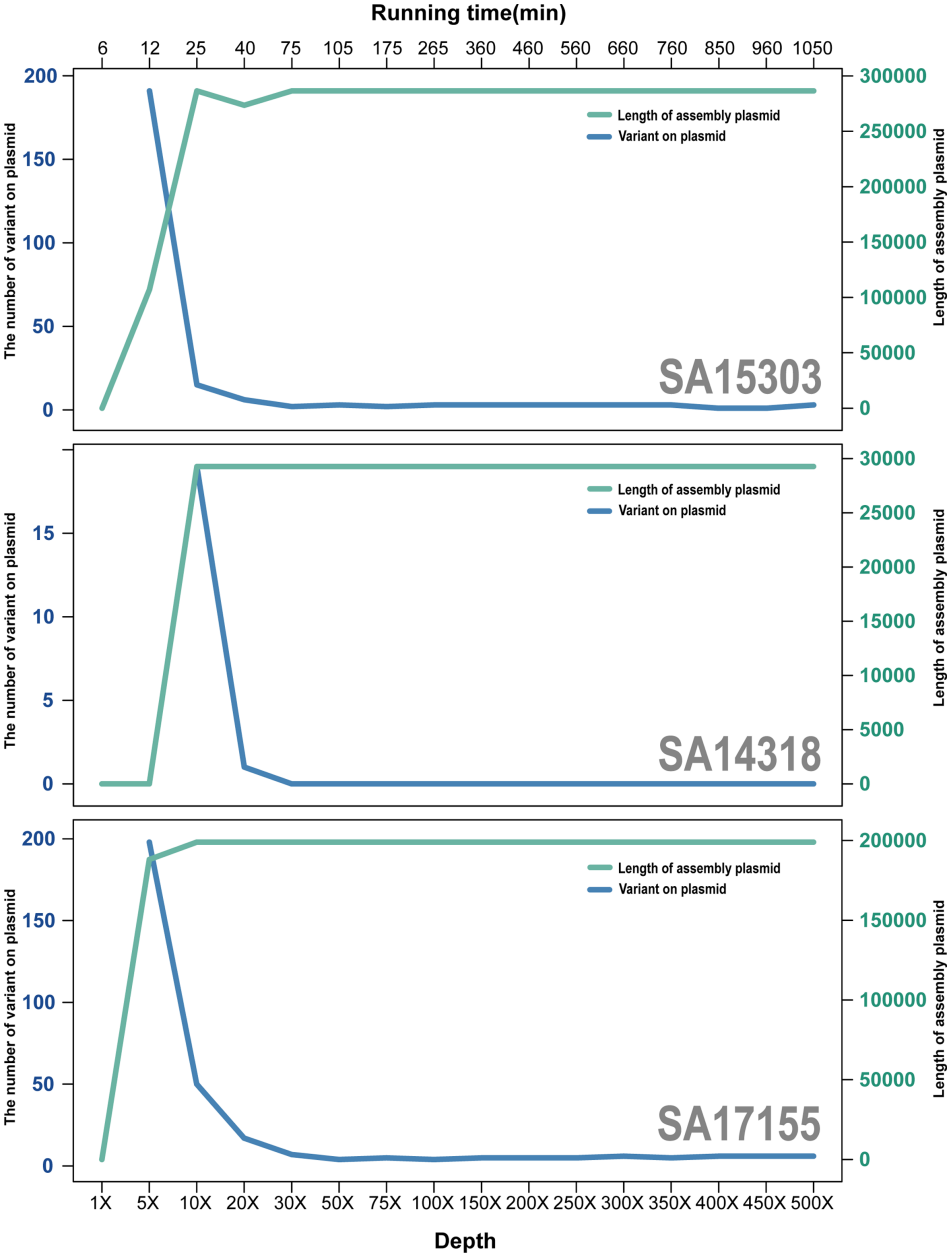
Trend plot of plasmid size and errors with increasing sequencing depth. Green lines and axes represent the size of plasmids, and blue lines represent the number of errors.

When the depth of sequencing data reached 75× coverage, the quality of the assembled sequences was near optimal. The average nucleotide identity was >99.9975% between each assembled sequence and the reference genome (Figure 4).

Compared with sequences at 75× coverage depth, chromosomal sequences at 500× coverage depth had an average error reduction of 1.67, whereas plasmids had an average error increase of 0.67. Thus, although the 500× sequencing required a further 875 min and generated 6.5-fold more data than the 75× sequencing, the effects were not noticeably improved.

The latest ONT kit SQK-LSK114 and flow cell R10.4.1 have dramatically reduced errors in the assembled genomes. For data coverage that exceeded 75× (750 Megabyte, 160 min), using only Nanopore sequencing data in *de novo* genome packing, complete sequences were achieved that nearly matched the accuracy of NGS without requiring short-read data.

## 4. Discussion

The spread of ARGs carried by plasmids is a major public health issue (San Millan, 2018). As mobile genetic elements that can carry ARGs and be transferred easily between different bacterial species, plasmids enable the quick and effective dissemination of ARGs (Vit et al., 2020). The emergence of MDR and the spread of drug resistance between bacterial strains can both be aided by plasmids (Paterson and van Duin, 2017). It is critical to comprehend how bacteria acquire and spread resistance to antibiotics, in addition to the molecular mechanisms of this phenomenon (Jordt et al., 2020). High-quality finished genomes of drug-resistant bacteria are required to monitor the transmission of antimicrobial resistance. The current tools for ARG detection are known to be highly accurate when used with short-read sequencing data. However, short-read sequencing cannot provide accurate localization of ARGs, which can be carried by both bacterial plasmids and chromosomes. More frequent cross-host resistance epidemic events initiated by plasmids carrying ARGs have occurred (Li et al., 2022), suggesting the greater potential for ARG transmission through plasmids in humans, food, animals, and the environment. To obtain high-quality finished genomes, it has been necessary to include short-read polishing of long-read sequencing data to correct errors (Senol Cali et al., 2019). This combined approach required two sequencing libraries and was time-consuming, difficult to perform, and can entail high costs. The optimal solution is to improve the accuracy of long-read sequencing. Based on long-read sequencing, one can locate ARGs on chromosomes or plasmids. Horizontal transfer of plasmids plays an important role in the spread of multidrug-resistant bacteria, identifying plasmid-borne resistance genes is necessary to estimate the spread of resistance among bacteria. The coexistence of ARGs is very common, and poses significant public health and food safety threats. Obtaining complete plasmid sequences is the most effective means of detecting ARGs and the coexistence of ARGs on the same plasmid.

In this study, the latest ONT SQK-LSK114 kit with flow cell R10.4.1 was used, and the results demonstrated that a high-quality finished genome could be obtained using *de novo* assembly. Compared to the previous process, the Q-score of reads was significantly improved (Smith et al., 2020). In previous studies, the limitation of ONT sequencing was the relatively high error rate, which in some cases can reach 10% (Khrenova et al., 2022). Low base-call accuracy has limited the reliability of ONT data for critical genomic epidemiology tasks such as ARG and virulence gene detection and typing, serotype prediction, and cluster identification (Foster-Nyarko et al., 2023). In this study, the average read accuracy was 98.9%, indicating that the novel approach has the potential to greatly improve read accuracy. When using the latest ONT SQK-LSK114 kit and R10.4.1 flow cell for long-read sequencing, error correction using short-read sequence data is not required. The novel ONT sequencing method saves money and time. Both the preparation of short- and long-read libraries as well as sequencing, which take at least 1 day, are unnecessary.

Regarding the new ONT sequencing method, once low-quality reads had been removed, the accuracy of the assembled sequences was nearly identical to that based on NGS. However, homopolymer regions increased the possibility of INS and DEL errors during assembly, and biased SNV errors still needed to be improved. In the future, the analysis pipeline may become more streamlined and effective.

In conclusion, use of the ONT LSK114 kit combined with flow cell R10.4.1 improved the accuracy of *de novo* genome assembly. When sequencing bacteria and plasmid, high-quality complete genomes with ideal coverage and identity were obtained without short-read or reference polishing. To obtain saturated raw data, we recommend acquiring a depth of 100× raw data (1 Gigabytes, 265 min), and performing error correction three times after assembly. This method will save time in obtaining high-quality complete genomes of bacteria for antimicrobial resistance surveillance, and is likely to become a valuable tool for monitoring the transmission of plasmid-borne drug resistance genes (Peter et al., 2020).

## Data availability statement

The datasets presented in this study can be found in online repositories. The names of the repository/repositories and accession number(s) can be found in the article/Supplementary material.

## Author contributions

XL, ZL, and BK conceived and designed this study. BP, ML, YP, and JX contributed to the experiment. WZh and WZe contributed to writing the manuscript. All authors contributed to the article and approved the submitted version.

## Funding

This work was supported by the National Key Research and Development Program of China (2020YFE0205700 and 2022YFC2303900), the Major Projects of the National Natural Science Foundation of China (22193064), and the Science Foundation (2022SKLID303) of the State Key Laboratory of Infectious Disease Prevention and Control, China.

## Conflict of interest

The authors declare that the research was conducted in the absence of any commercial or financial relationships that could be construed as a potential conflict of interest.

## Publisher’s note

All claims expressed in this article are solely those of the authors and do not necessarily represent those of their affiliated organizations, or those of the publisher, the editors and the reviewers. Any product that may be evaluated in this article, or claim that may be made by its manufacturer, is not guaranteed or endorsed by the publisher.

## Footnotes

^1^ https://github.com/nanoporetech/medaka

^2^ https://github.com/tseemann/snippy
