# Supplementary material for: Oxford nanopore long-read sequencing enables the generation of complete bacterial and plasmid genomes without short-read sequencing
Source: Front Microbiol. 2023 May 15;14:1179966. doi: 10.3389/fmicb.2023.1179966 (PMC10225699; doi:10.3389/fmicb.2023.1179966)
Supplement: Supplementary file 1 [file Table_1.DOCX]

**Supplementary Table 1 Results of error correction with multiple correction by Medaka**

| Rawdata size(Megabyte) | | 16.86 | 43.40 | 102.01 | 176.98 | 328.38 | 526.00 | 764.60 | 1.14×10^3^ | 1.61×10^3^ | 2.05×10^3^ | 2.49×10^3^ | 2.93×  10^3^ | 3.37×10^3^ | 3.82×10^3^ | 4.26×10^3^ | 4.73×10^3^ |
| --- | --- | --- | --- | --- | --- | --- | --- | --- | --- | --- | --- | --- | --- | --- | --- | --- | --- |
| Depth(×) | | 1 | 4 | 10 | 18 | 33 | 53 | 78 | 120 | 169 | 215 | 261 | 308 | 353 | 402 | 448 | 497 |
| Whether it is circular(chromosome/plasmid) | | N/N | N/N | N/Y | Y/Y | Y/Y | Y/Y | Y/Y | Y/Y | Y/Y | Y/Y | Y/Y | Y/Y | Y/Y | Y/Y | Y/Y | Y/Y |
| Before correction | Variant-DEL | 12 | 365 | 68 | 18 | 11 | 13 | 10 | 13 | 9 | 10 | 11 | 11 | 10 | 9 | 7 | 10 |
|  | Variant-INS | 54 | 2006 | 312 | 25 | 6 | 2 | 3 | 1 | 1 | 1 | 3 | 1 | 0 | 0 | 0 | 1 |
|  | Variant-SNP | 27 | 1210 | 174 | 13 | 5 | 2 | 3 | 4 | 4 | 4 | 4 | 4 | 5 | 7 | 7 | 7 |
|  | VariantTotal | 93 | 3581 | 554 | 56 | 22 | 17 | 16 | 18 | 14 | 15 | 18 | 16 | 15 | 16 | 14 | 18 |
| After one correction | Variant-DEL | 29 | 989 | 136 | 6 | 2 | 2 | 2 | 2 | 2 | 2 | 2 | 2 | 2 | 2 | 2 | 2 |
|  | Variant-INS | 43 | 1509 | 191 | 16 | 8 | 8 | 7 | 6 | 7 | 5 | 4 | 5 | 4 | 2 | 5 | 4 |
|  | Variant-SNP | 36 | 1374 | 149 | 9 | 3 | 2 | 2 | 2 | 2 | 2 | 2 | 2 | 2 | 2 | 2 | 2 |
|  | VariantTotal | 108 | 3872 | 476 | 31 | 13 | 12 | 11 | 10 | 11 | 9 | 8 | 9 | 8 | 6 | 9 | 8 |
| After three corrections | Variant-DEL | 25 | 999 | 139 | 6 | 2 | 2 | 2 | 2 | 2 | 2 | 2 | 2 | 2 | 2 | 2 | 2 |
|  | Variant-INS | 42 | 1477 | 185 | 17 | 8 | 8 | 7 | 6 | 7 | 5 | 4 | 5 | 4 | 2 | 5 | 4 |
|  | Variant-SNP | 35 | 1423 | 159 | 9 | 2 | 2 | 2 | 2 | 2 | 2 | 2 | 2 | 2 | 2 | 2 | 2 |
|  | VariantTotal | 102 | 3899 | 483 | 32 | 12 | 12 | 11 | 10 | 11 | 9 | 8 | 9 | 8 | 6 | 9 | 8 |
