# Supplementary material for: Oxford nanopore long-read sequencing enables the generation of complete bacterial and plasmid genomes without short-read sequencing
Source: Front Microbiol. 2023 May 15;14:1179966. doi: 10.3389/fmicb.2023.1179966 (PMC10225699; doi:10.3389/fmicb.2023.1179966)
Supplement: Supplementary file 2 [file Table_2.DOCX]

**Supplementary Table 2 Location of deletion (DEL) and insertion (INS) errors for each genome**

| ID | Accession | POS | TYPE | REF | ALT |
| --- | --- | --- | --- | --- | --- |
| SA15303 | SAMN33419349 | 645781 | DEL | GCAAAAAAAAAAAA | GC-AAAAAAAAAAA |
|  |  | 2552758 | INS | ATTTTA-TTTTTC | ATTTTATTTTTTC |
|  |  | 4330412 | INS | C-GGGGGGGGGGGG | CGGGGGGGGGGGGG |
| SA14318 | SAMN33419350 | 1155739 | INS | A-GGGGGGGG | AGGGGGGGGG |
|  |  | 1515807 | INS | A-TTT | ATTTT |
|  |  | 2658706 | INS | T-CCCCCCCCC | TCCCCCCCCCC |
| SA17155 | SAMN33419351 | 4350144 | INS | A-AG | AGAG |
|  |  | 853760 | INS | ATTTTA-TTTTT | ATTTTATTTTTT |
|  |  | 3880661 | DEL | CAAAAAAAAAAAA | C-AAAAAAAAAAA |
