# Supplementary material for: Oxford nanopore long-read sequencing enables the generation of complete bacterial and plasmid genomes without short-read sequencing
Source: Front Microbiol. 2023 May 15;14:1179966. doi: 10.3389/fmicb.2023.1179966 (PMC10225699; doi:10.3389/fmicb.2023.1179966)
Supplement: Supplementary file 3 [file Presentation_1.PDF]

Supplementary Code: R Script for Data Analysis

```
## This code uses R to analyze experimental data, including data preprocessing, statistical analysis.

# rawdata to cleandata
dir("./rawdata")->mmm
dir.create("cleandata")
system.time(parLapply(cl,mmm,function(i){
code <-sprintf("gunzip -c ./%s | NanoFilt -q 10 -l 1000 --headcrop 50 --tailcrop    50|
gzip > ./cleandata/%s",i,i)
system(code)}))

# Assemble the reference genome using long-read and short-read data
system("nohup unicycler -1 ./sa17155_1.fq.gz -2 ./sa17155_2.fq.gz -l nanoplore.gz -o ref &")

# Use Flye to assemble the genome with only long-read sequencing
system("software/Flye/bin/flye -o ./flye/ -i 10 -t 8 -g 5m --nano-raw ./nanoplore.fq --plasmids")

# Polish the genome using Madaka
code= ("medaka_consensus -i ./nanoplore.fq -d ./assembly.fasta -o ./medaka_output -t 4 -m
r103_min_high_g360")
system(code)

# Use BLAST to get the identity of the fastq file compared to the reference genome
system("fastq_to_fasta -i nanopore.fastq -o nanopore.fasta")
system("makeblastdb -i sa17155_ref.fa -dbtype nucl -o ref")
system("blastn -query nanopore.fasta -db ref    -out nanopore.tab -outfmt '6' ")

# Use fastANI to get the identity of assemble.fasta compared to the reference genome
system("fastANI -q assemble.fasta.fa -r sa17155_ref.fa -o output.txt")

# Use snippy to compare assemblefasta to a reference genome. Get the number of SNPs, INSs,
DELs
system("snippy --outdir 130.fq --ctgs ./assemble.fasta --ref ./sa17155_ref.fa ")
```
